# Case Report: Clinical, laboratory, and pathological findings in cows with osteomyelitis of the ribs and sternum and endocarditis valvularis thromboticans

**DOI:** 10.3389/fvets.2025.1589472

**Published:** 2025-07-11

**Authors:** Fanny Rachidi, Abass Elhadi, Lilli Bittner-Schwerda, Melanie Schären-Bannert, Gábor Köller, Jan Schinköthe, Anna Majcher, Benjamin Diehl, Florian Hansmann, Tilman Kühn, Alexander Starke

**Affiliations:** ^1^Clinic for Ruminants and Swine, Faculty of Veterinary Medicine, Leipzig University, Leipzig, Germany; ^2^Department of Surgery and Anaesthesia, Faculty of Veterinary Medicine, University of Khartoum, Khartoum, Sudan; ^3^Faculty of Veterinary Medicine, Institute of Veterinary Pathology, Leipzig University, Leipzig, Germany

**Keywords:** osteomyelitis, endocarditis, *Trueperella pyogenes*, bacteriology, blood culture, ultrasonography, postmortem examination, cattle

## Abstract

The etiological relationship between osteomyelitis (OM) of the ribs and sternum and endocarditis valvularis thromboticans (EVT) in dairy cattle was assessed using clinical, ultrasonographic, bacteriologic, and postmortem findings. Five dairy cows (2nd–6th lactation, 135–304 days in milk, 490–630 kg) were admitted to our clinic because of low production and poor body condition. Clinical examination revealed abnormalities in the circulatory and locomotor system, as well as the ribs and sternum. Ultrasonographic examinations of the heart, ribs, and sternum were performed, and samples were collected for laboratory analyses. The cows underwent an orthopedic examination and bacteriologic testing of blood (*N* = 5), abscesses of the ribs or sternum (*N* = 3), and synovial fluid (*N* = 2). All five cows were euthanized because of a poor prognosis, and a postmortem examination was carried out. During the postmortem examination, samples were collected from endocardial lesions (*N* = 4) and abscesses of the ribs and/or sternum (*N* = 2) that had not been accessible *intra vitam*. The physical condition of the cows suggested a chronic metastatic disease accompanied by pyemia. All cows had OM of the ribs or sternum and EVT, along with other inflammatory diseases, including arthritis (*N* = 4), tendovaginitis (*N* = 2), and abscesses (*N* = 2). These clinical diagnoses were confirmed during the postmortem examination. Bacteriologic examination revealed *Trueperella pyogenes* in the rib and sternal lesions (*N* = 5), blood cultures (*N* = 3), endocardial lesions (*N* = 3), and samples taken from the uterus, kidney, spleen, and muscle (*N* = 4). Our results emphasize the importance of ruling out EVT in cattle with lesions of the ribs and sternum. Ultrasonographic and microbiologic examinations support the diagnosis and help determine the extent and severity of the changes. While a definitive primary cause cannot be identified due to the animals’ polymorbid condition, the observed lesions suggest that apostematous inflammation of the ribs or sternum may act as a potential source of infection.

## 1 Introduction

Rib and sternal lesions in cattle have been observed at the herd level (1–3), in abattoirs (4–7), and during postmortem examination (8, 9). While only externally visible rib swellings have been described at the herd level (2), rib and sternal fractures (5, 7, 9–17), inflammation of the costochondral junction (CCJ) (4, 18, 19), and osteomyelitis (OM) (6, 11, 19) have been documented at the slaughterhouse and during postmortem examinations. In adult cattle, they are assumed to be caused by trauma (20) and various technopathies, especially in tie-stall systems (21). Lame cows may lie down too quickly or awkwardly on poorly designed surfaces (12). Inadequate bone mineralization (16), osteoporosis (17), and behaviors associated with the establishment of social hierarchy within a herd (15) may also play a role. In addition, rib fractures in calves can result from trauma at birth (8, 13). Risk factors for rib fractures in adult cattle include lameness (1, 5, 22, 23), increasing age (1, 5, 23), malnutrition (16, 17), increased calcium mobilization in late pregnancy (14), narrow passageways, and inadequate feed bunk space and cubicle design (1). Rib and sternal lesions can lead to pain and impairment of well-being, behavior, and performance, as well as life-threatening concomitant diseases such as endocarditis valvularis thromboticans (EVT) (10, 11, 14, 15, 18). Although apostematous inflammation of the ribs or sternum and life-threatening concomitant diseases associated with bacteremia have been reported, a potential etiology linking these lesions has not yet been investigated (PubMed search on 12 April 2025, keywords: bacteremia, rib, sternum, adult cattle).

This case series describes five cows with EVT and osteomyelitis of the ribs and/or sternum, aiming to identify the etiology of and relationship between rib and sternal lesions and EVT using clinical, laboratory, diagnostic imaging, and postmortem findings.

## 2 Case presentation

### 2.1 Animals and diagnostic procedures

Between 2018 and January 2024, 2,611 cattle were examined at the Clinic for Ruminants and Swine, Faculty of Veterinary Medicine, Leipzig University, and 6,210 animals were evaluated during on-farm visits. Rib abnormalities were documented in 284 cows, of which 66 underwent detailed diagnostic evaluation. EVT was confirmed in 16 of these cases. This case series describes five German Holstein cows (median age 5.5 years…-304) selected from the 16 EVT-positive cows across five different dairy farms. All five cows were at least in their first lactation and had rib or sternal lesions and EVT confirmed by both ultrasonography (performed by the first and last authors) and postmortem examination. The cows were pre-treated by the on-farm veterinarians with anti-inflammatory drugs and antibiotics (*N* = 5), an intravenous infusion of 0.9% NaCl solution (*N* = 1), and a ruminal magnet (*N* = 1). They were admitted to our clinic due to poor production performance and body condition (*N* = 5), recurrent fever (*N* = 2), and abortion (*N* = 1).

The cows underwent thorough clinical (24) and ultrasonographic (25–28) examinations, including lesion assessment and echocardiography. Blood, aspirated fluid, and tissue samples were collected for hematologic, clinical chemistry, and microbiologic analyses. The owners were contacted immediately after the diagnoses were established (in the case of cow #4, after the follow-up examination), and euthanasia was recommended due to the poor prognosis. After euthanasia, a complete postmortem examination was conducted, including tissue fixation and histologic evaluation. A detailed description of the diagnostic procedures is provided in the Supplementary material.

### 2.2 Results

#### 2.2.1 Clinical findings

At the time of admission, all five cows had a mildly to moderately abnormal general condition, mild lameness, and moderate to poor body condition. The heart rate was higher than the reference range (60–90 beats per min) (24) in four cows, the respiratory rate was elevated in two cows (reference range: 24–36 breaths per min) (24), and the rectal temperature was increased in one cow (reference range: 38–39°C) (24). The general physical condition of all cows suggested a chronic disease process. Based on the results of the clinical examination, abnormalities in the circulatory and musculoskeletal systems were tentatively diagnosed (Supplementary Table 2).

The clinical examination of the circulatory system revealed congested jugular veins, tachycardia, and abnormal heart sounds (systolic endocardial murmur) in three cows. In all three cases, the point of maximal audibility was located in the area of the tricuspid valve. Cow #5 also showed arrhythmia. In cow #4, initial findings of the circulatory system were unremarkable except for a slightly elevated heart rate; however, a follow-up examination 2 months later showed significant cardiovascular deterioration, loss of body condition, and poor performance (Supplementary Table 2). For the other four cows, a long-term follow-up was not feasible due to animal welfare considerations.

**Figure 1:**
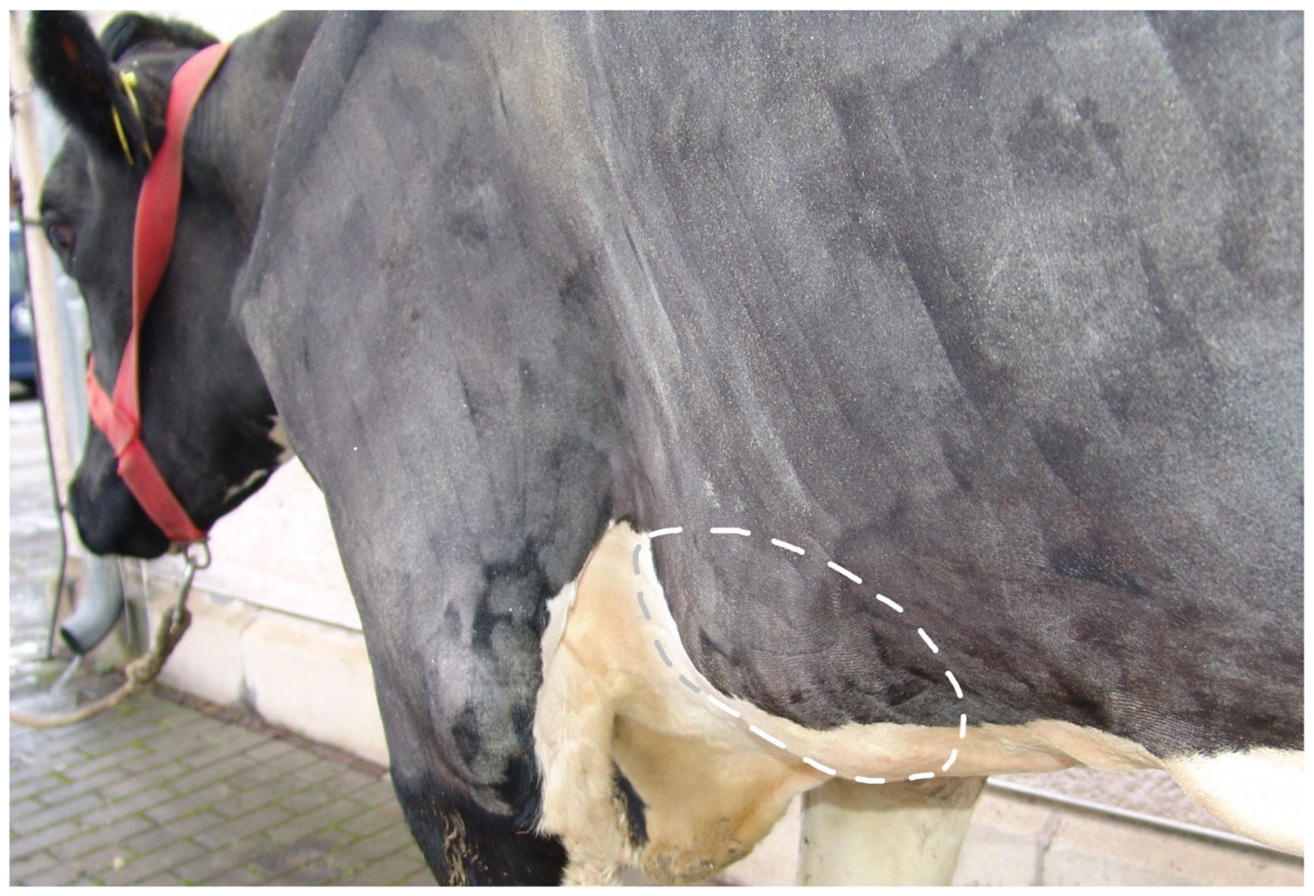
Image of a 4.5-year-old pregnant German Holstein cow (#2, 2nd lactation, 287 days in milk) diagnosed with endocarditis valvularis thromboticans. Caudolateral view from the left side showing the extensively clipped thorax and forelimb. The dashed circle shows an approximately 30 × 20 × 4 cm swelling associated with the 5th to 7th ribs, including the corresponding sternebrae. The skin over the swelling was intact.

The rib and sternal lesions appeared as round to oval swellings without visible skin changes (Figure 1). The five cows had 15 abnormalities in the ribs (*N* = 11) or in both the ribs and sternum (*N* = 4). The lesions primarily involved the 8th to 10th ribs and the adjacent sternal region; in cow #2, the 5th to 7th ribs were affected, and in cow #5, the 13th rib was involved in addition to the 8th to 10th ribs. They were located at the level of the elbow (*N* = 6), the sternum (*N* = 3), and the shoulder joint (*N* = 2). The lesions were mostly bilateral, non-mobile, predominantly bony, without crepitation, and did not feel warmer than normal. Pressure elicited a pain response in three cows (Supplementary Table 2).

The examination of the musculoskeletal system revealed lameness in all cows (grade I to III out of V) (29), with joint abnormalities (*N* = 3), tendon sheath abnormalities (*N* = 2), or abscesses in the musculature (*N* = 2; Supplementary Table 2).

#### 2.2.2 Further diagnostics

Laboratory testing revealed increased inflammatory markers, including leukocytosis, increased total protein, decreased albumin concentration, and a positive glutaraldehyde test. In cow #4, the results were outside the reference ranges at both the initial and follow-up examinations (Supplementary Table 3).

Sonographic examination of the heart revealed hyperechoic, circumferential, cauliflower-like enlargement of the tricuspid valve (*N* = 5) and bicuspid valve (*N* = 3). The pericardium, epicardium, and myocardium were unremarkable. In cow #4, no cardiac abnormalities were observed at the initial examination, but bicuspid and tricuspid valves were abnormal at the follow-up examination 2 months later.

**Figure 2:**
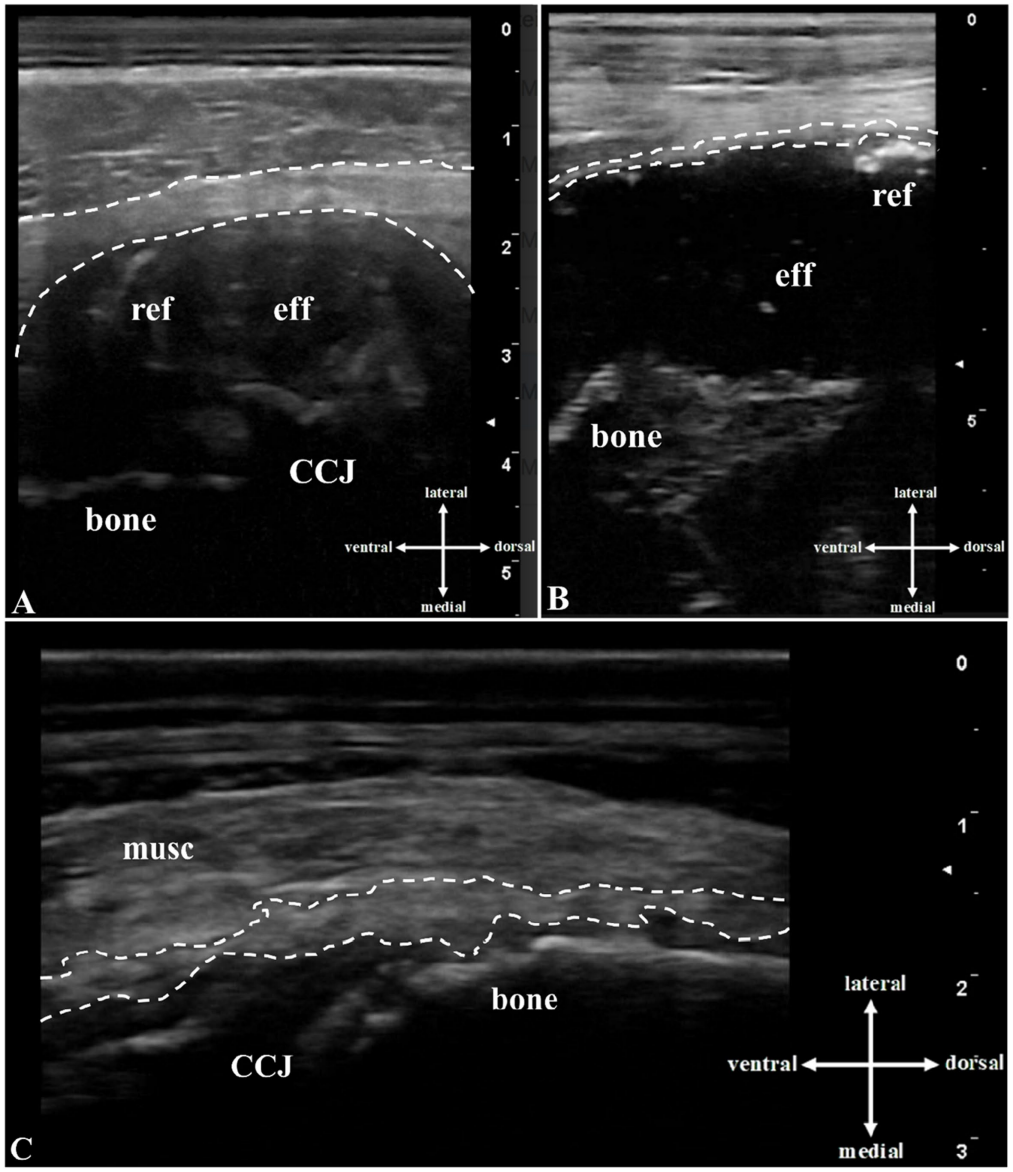
Transcutaneous B-mode ultrasound image (10-MHz linear transducer, penetration depth 3 to 8 cm) of the rib lesions: In the area of the **(A)** left 4th rib of cow #1: A hypoechogenic, fluid-filled cavity (eff) lateral to the uneven echogenic bone (bone), echogenic reflexes (ref), and an echogenic capsule (space between dashed lines) are seen above the costochondral junction (CCJ). **(B)** In the area of the left 7th rib of cow #2: A hypoechogenic, fluid-filled cavity (eff) lateral to the irregularly-shaped echogenic bone, echogenic and hyperechogenic reflexes (ref), and an echogenic capsule (space between dashed lines) are evident. **(C)** In the area of the left 8th rib of cow #5: The musculature is thicker than normal and irregular, with hyperechogenic and hypoechogenic areas (musc). Thickened, elevated echoic periosteum (space between dashed lines) and hyperechogenic irregular bone with hyperechogenic exostoses (bone) near the costochondral junction (CCJ) are present.

The transcutaneous ultrasonography of the ribs and sternum showed unremarkable skin but thickened, irregular musculature with hypoechogenic and hyperechogenic areas in four cows (Figure 2). In all cows, the periosteum was detached from the bone, which appeared irregular and, in two cows, showed step-like interruptions. Widened costochondral junctions were seen in four cows, with exostoses, axial deviations, and fluid-filled cavities in three. These cavities had an echogenic capsule and contained hypoechogenic to anechogenic fluid with echogenic reflexes. Flow phenomena were visualized in two cases (*N* = 2), and ultrasound-guided aspiration yielded creamy, light yellow exudate in three cases (*N* = 3; Table 1).

**Table 1:** Results of the ultrasonographic examination of the ribs in the five cows with swellings associated with the ribs or sternum and endocarditis valvularis thromboticans.

Cow	#1	#2	#3	#4	#5
Region	8th rib & sternum (L)	5th – 7th rib (L)	9th rib & sternum (L)	8th rib (L & R)	8th – 10th rib (L) 8th, 9th, 13th rib (R)
Soft tissue	Skin NAD, surrounding musculature thickened and irregular with hyperechogenic and hypoechogenic areas, fluid-filled cavity with approx. 0.5 cm echogenic capsule and hypoechogenic border lateral to the bone	Skin and musculature NAD, fluid-filled cavity lateral to the bone with indistinct echogenic demarcation from surrounding tissue	Skin and musculature NAD, fluid-filled cavity with indistinct demarcation from surrounding tissue and lateral to the bone line	Skin NAD, surrounding musculature thickened and heterogeneous with hypoechogenic areas, fluid-filled cavity with indistinct demarcation from surrounding tissue and lateral to the bone line	Skin NAD, heterogeneous hypoechogenic areas in subcutaneous tissue, musculature thickened and hyperechogenic with irregularly distributed hypoechogenic areas
Periosteum	Detached from the bone	Detached from the bone	Detached from the bone	Detached from the bone	Detached from the bone
Bone surface	Uneven, interruptions in the contour creating a step-like appearance	Irregular contour, interruptions creating a step-like appearance	Uneven with irregular contour	Uneven with an irregular appearance and hyperechogenic exostoses	Uneven with irregular contour
Costochondral junction	Enlarged, with hyperechogenic exostoses, axial deviation, step-like appearance with heterogeneous echogenicity	Enlarged, with a fluid-filled cavity laterally	NAD	Enlarged, with a fluid-filled cavity	Enlarged, with a hyperechogenic line close to the transducer, a heterogeneous echogenic appearance medial to the transducer
Effusion	Hypoechogenic with numerous echogenic reflexes, partly with shadow artifacts	Hypoechogenic with numerous echogenic and hyperechogenic reflexes	Hypoechogenic with scattered hyperechogenic reflexes	Anechogenic with numerous, irregularly distributed, floating, hyperechogenic reflexes of various sizes	Not present
Sonopalpation	Flow phenomenon could not be induced	Flow phenomenon	Flow phenomenon could not be induced	Flow phenomenon	
Ultrasound-guided centesis	Effusion light yellow and creamy	Effusion light yellow and creamy	Effusion content could not be aspirated	Effusion light yellow and creamy	

NAD, nothing abnormal detected; R, rib.

The results of the bacteriologic testing of the aspirate, blood culture, and various tissues are shown in Supplementary Table 4. *Trueperella pyogenes* was detected in 18 of the 21 positive microbiologic tests. The pathogen was identified in all rib and sternal lesions, in 60% of the blood cultures examined, 75% of the analyzed endocardial lesions, and in all other sampled organs or lesions (stifle joint, tendon sheath, uterus, spleen, kidney, and abscesses). In cow #4, *Trueperella pyogenes* was cultured from fluid aspirated from the cavity near the rib at both the initial and follow-up examinations. In addition, the enriched blood culture medium tested positive for *Helcococcus ovis*.

### 2.3 Clinical diagnosis and outcome

Based on the clinical and ultrasonographic findings, all cows were diagnosed with EVT. Septicemia and osteomyelitis (OM) of the ribs (*N* = 3) or both the ribs and sternum (*N* = 2) were tentatively diagnosed based on the clinical findings. In addition, arthritis (*N* = 4), tendovaginitis (*N* = 2), and abscess (*N* = 2) were diagnosed. In cow #4, there was no indication of EVT at the initial examination (clinical and ultrasonographic). Therefore, local treatment of claw disorders and abscesses in both hind limbs was performed, along with systemic antimicrobial and analgesic therapy. After the completion of the treatment, further monitoring was delegated to the owner. At the follow-up examination 2 months later, the cow’s condition had deteriorated, and in addition to the rib lesions and musculoskeletal disorders, EVT was diagnosed. Due to the guarded (*restitutio ad vitam*) or grave (*resitutio ad integrum*) prognosis, all cows were euthanized after consultation with the owners. In cow #4, euthanasia was performed at the time of the follow-up examination, whereas the remaining four cows were euthanized immediately after diagnosis at initial presentation. A postmortem examination was performed on all cows.

### 2.4 Gross and microscopic findings

**Figure 3:**
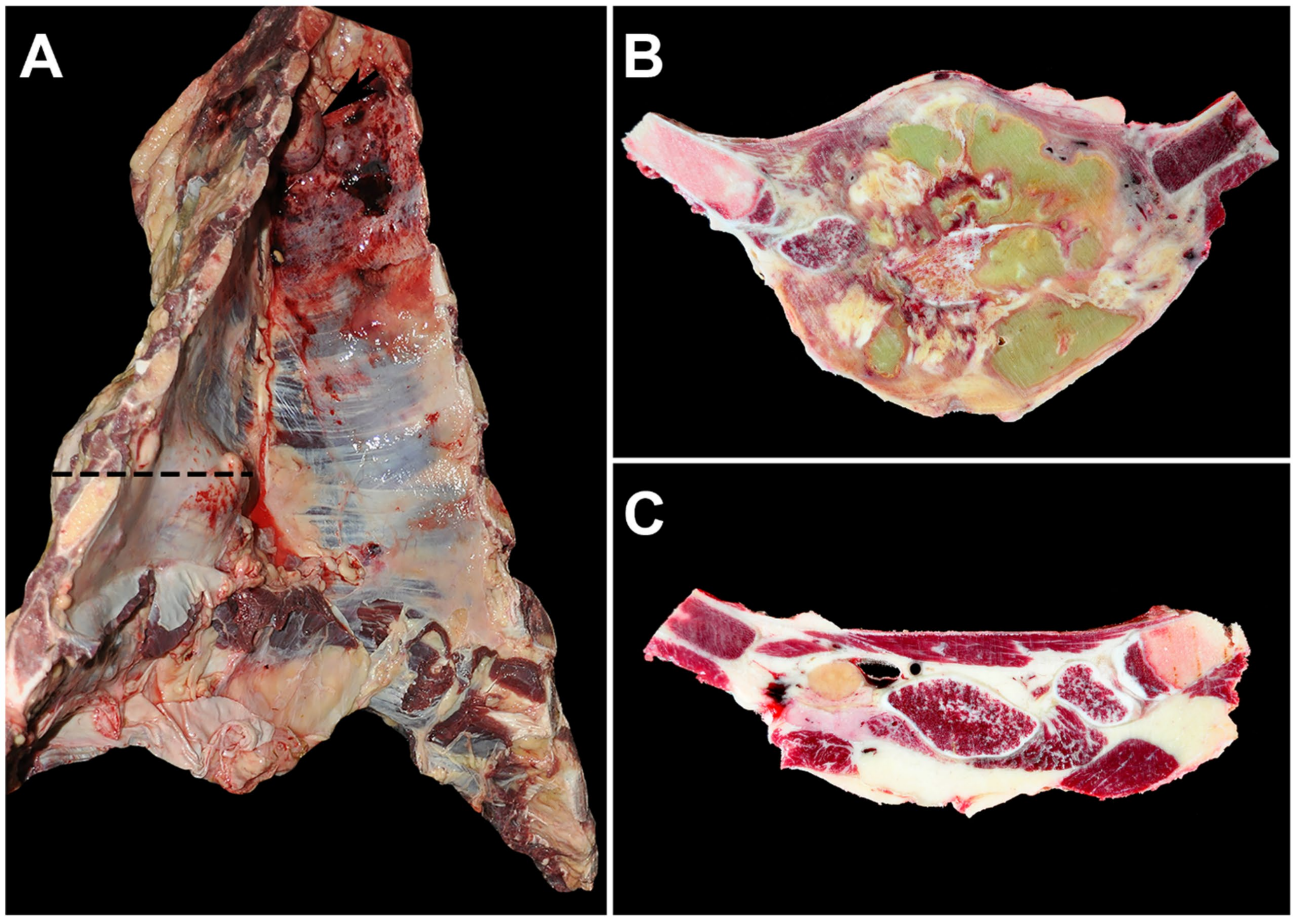
Thorax with severe lesions of the ribs and adjacent sternum (cow #2). **(A)** A space-occupying mass affecting the left 5th to 7th ribs and extending particularly far into the thoracic cavity (dashed line) with a severely enlarged cranial thoracic lymph node (arrow) is visible. **(B)** A cross-section of the tissue along the dashed line in image **A** reveals the mass as severe, chronic, multifocal to confluent, suppurative osteomyelitis and costochondritis with multifocal abscesses. **(C)** For comparison, the same sectional plane as in image **B** shows unaltered ribs and musculature on the right side of the thorax.

All cows had suppurative to pyogranulomatous OM and costochondritis in at least one rib, with cow #5 having six affected ribs (Supplementary Table 5). Cow #2 had a large space-occupying extra-and intra-thoracal lesion involving the ribs and sternum that measured up to 17.5 cm (craniocaudal) × 12.5 cm (lateromedial) × 17.5 cm (dorsoventral, Figures 3A,B). The affected areas showed clearly visible macroscopic changes compared to unaffected regions of the thorax (Figure 3C).

The cows had lesions suggestive of embolic showering, frequently observed as EVT in bicuspid and tricuspid valves. Severe lesions were seen in the tricuspid valve (Supplementary Figure 1A), in contrast to the mild to moderate lesions observed in the bicuspid valve. Cow #5 had EVT involving the aortic valve. Granulation tissue formation from the adjacent mural endocardium and myocardium with septic emboli containing numerous coccoid bacteria enmeshed in degenerating neutrophils and fibrin clots was observed. Septic embolic showering was also evident as suppurative nephritis (*N* = 5, Supplementary Figure 1B), vasculocentric suppurative pneumonia (*N* = 4), and suppurative splenitis (cow #1; Supplementary Figure 1D).

The musculoskeletal system, particularly the joints, was also affected (Supplementary Table 5). Moderate to severe suppurative to fibrin-suppurative arthritis was prominent in the majority of the cows (*N* = 4), frequently affecting the stifle joints (Supplementary Figure 1C). Cow #4 had severe suppurative myositis with multifocal abscesses in the semitendinosus and semimembranosus muscles. Inflammation of the skin, subcutis, bone, and claw wall was observed in the hind claws of three cows (Supplementary Table 5). Other lesions, which in all likelihood were not embolic, included suppurative mastitis (cow #1), ulcerative dermatitis of the sternal region (cow #2), and moderate suppurative endometritis (cow #5). In addition, in three cows, mild to moderate, multifocal lymphohistioplasmacytic to suppurative meningoencephalitis was seen.

## 3 Discussion

### 3.1 Diagnosis based on the results of the clinical and pathologic examinations

To the best of the authors` knowledge, OM of the ribs or sternum, bacteremia, and EVT have not been described in live cows (PubMed search on 12 April 2025, keywords: bacteremia, endocarditis, osteomyelitis, rib, sternum, cattle). In human medicine, septicemia and EVT have been reported as complications of sternal abscesses (30–35), but no cases describe EVT as the primary cause of rib or sternal OM. The disease course in cow #4 suggests a similar sequence: although the initial clinical and ultrasonographic examinations revealed no abnormalities of the cardiovascular system, EVT was diagnosed 2 months later based on both clinical signs and ultrasonographic findings. Whether a compensated EVT with lesions smaller than 0.5 cm, which may have been undetectable by ultrasonography (28), was already present at the time of the initial examination cannot be ruled out definitively. In contrast, the rib and sternal lesions were already evident during the first examination and remained unchanged. Although such longitudinal case tracking has not yet been conducted in cattle, the recommendation to examine the ribs and sternum in slaughtered cows suspected of bacteremia (4), highlighting a potential link between these lesions and bacteremia. Inflammatory changes in other bones have also been described as a cause of EVT in cattle (36–38).

Our results revealed that OM of the ribs and sternum occurred in the cattle with bacteremia and EVT. The course of disease in cow #4 suggests that OM of the ribs and sternum may have preceded the development of EVT and bacteremia. However, given the advanced and polymorbid condition of all cows in this series, a definitive conclusion regarding the temporal and causal relationship between these lesions and systemic infection cannot be drawn. A hematogenous spread originating from other, undetected infectious foci remains a possibility. On the other hand, a comprehensive postmortem examination of all organ systems was carried out in all cases, and such infectious foci would very likely have been identified. Therefore, we consider rib and sternal OM as potential contributors to systemic infection in cattle and recommend their inclusion in diagnostic evaluations, especially in animals presenting with signs of bacteremia or EVT.

### 3.2 Diagnostic imaging and laboratory tests

Diagnostic imaging and laboratory tests are essential for diagnosing these conditions. Hyperproteinemia, hypergammaglobulinemia, and leukocytosis are typical findings in cows with EVT (38). The glutaraldehyde test, a rapid test for identifying hypergammaglobulinemia (39), was positive in all cows. Chemical and microbiologic analyses of blood and exudate also provided valuable information for the diagnosis of EVT (40, 41) and OM of the ribs and sternum (10). Ultrasonography of the heart is recommended for the diagnosis of EVT in live cattle (42). Endocardial lesions exceeding 0.5 cm can be visualized using ultrasonography (28). In contrast, procedures for diagnosing OM of the ribs and sternum in live cattle are lacking (PubMed search on 12 April 2025, keywords: osteomyelitis, rib, sternum, cattle, ultrasonography). Although cattle with OM of the long bones often exhibit characteristic clinical findings (43), the signs of OM of the ribs and sternum are non-specific, and diagnosis by inspection and palpation alone is difficult (10). Palpation of lesions is limited to external examination, but in some cases, lesions can only be detected internally, such as during abdominal surgery. Diagnostic imaging, primarily ultrasonography and radiography, is required to diagnose OM of the long bones in cattle. Characteristic findings in cattle with OM, which we were also able to visualize in the abnormal rib and sternal areas (Figure 2; Table 1), are soft tissue swelling, thickening and detachment of the periosteum, and irregular rough bone contours that may have a step-like appearance (26). In foals and people with OM of the ribs and sternum, ultrasonography is considered a better diagnostic tool than radiography (44–47). However, ossification of abscess capsules or other structures, as seen in cow #5, can result in soft tissue structures being further away from the transducer and inaccessible because of sound cancelation (48). In all five cows, the swellings associated with the ribs and sternum, which initially appeared benign on clinical examination, turned out to be merely the tip of the iceberg. Therefore, when similar abnormalities are found during clinical examination, a thorough evaluation of the patient, including the cardiovascular system, should be carried out using the diagnostic methods described above. Ultrasonography is particularly useful, providing important information that usually contributes significantly to the diagnosis and prognosis.

### 3.3 Microbiology

*Trueperella pyogenes* was identified as the main pathogen in all cases, reinforcing its role in purulent infections such as EVT (40, 49) and OM (43, 50) in cattle. *Trueperella pyogenes* was reported in only one cow with EVT and abscessation of the costochondral junction of the 3rd rib; however, blood culture was not performed (18). While blood culture has been used to diagnose EVT in cattle (37), it has not been a diagnostic tool for OM (PubMed search on 12 April 2025, keywords: osteomyelitis, blood culture, cattle). Findings in humans with OM of the ribs and sternum show that bacteremia and subsequent EVT occur, and early treatment, facilitated by microbiological diagnostics, can halt disease progression (30). When performed correctly, microbiologic testing is a valuable diagnostic tool. In the present report, conventional sampling, storage, and culture techniques were used successfully. Proper execution of these steps and the timing of collection are crucial (51). In agreement with other studies, the conventional blood culture system used in the present study was effective for blood analysis (52, 53), and Amies medium sample tubes were the appropriate choice for exudates, synovial fluid, and small tissue samples (51). In line with the recommendations for cattle with arthritis and concurrent OM (54), we collected tissue samples rather than synovial fluid for the microbiologic testing. In contrast, there are currently no standardized guidelines for the collection and analysis of blood cultures in cattle, horses, or companion animals (55). In our study, two cows had negative blood culture results. Given that all animals had received antibiotic treatment prior to referral, the absence of detectable pathogens in these cases may have been due to antimicrobial interference or intermittent bacteremia. However, prior antimicrobial therapy should not be considered a contraindication for blood cultures, as repeated sampling and increased blood volume can enhance detection rates even under such conditions (56, 57). In human medicine, when a bloodstream infection is suspected, it is recommended to collect two to four blood culture sets, each containing 20 to 30 mL of blood, ideally obtained several hours apart. In cases of endocarditis, even more frequent blood culture sampling may be necessary (56, 57). However, both approaches require considerable time and financial resources and must be weighed against the clinical benefits in routine settings (55, 58). In our cases, it was not feasible to collect a minimum of three blood cultures at one-hour intervals, as recommended for cattle with EVT (59). To compensate, we collected both aerobic and anaerobic blood cultures from each cow, effectively doubling the total blood volume analyzed. Given that *Trueperella pyogenes* can grow in both aerobic and anaerobic media, this approach likely improved the diagnostic yield. The isolation of *Trueperella pyogenes* from the blood cultures, endocardial lesions, and fluid-filled cavities associated with the ribs and sternum suggested a possible link between these abnormalities. Further studies are needed to determine the prevalence of this disease process on a larger scale, the involvement of other pathogens, and the potential virulence factors contributing to the development of OM of the ribs and sternum in cattle infected with *Trueperella pyogenes*.

### 3.4 Potential causes

Despite originating from different dairy farms with varying stall designs and levels of cow comfort, all cows exhibited musculoskeletal disorders and lameness in addition to EVT and OM. While lameness prevalence ranged from 24 to 60% across the farms (Supplementary Table 1), only individual affected cows were presented for examination. Although lameness prevalence varied across the herds and appeared relatively high, particularly on farm 5, the recorded rates remained within the typical range reported for dairy farms in the region (60, 61). No prior trauma had been reported in any of the animals, and there were no skin changes over the rib and sternal lesions. Interestingly, skin changes were also absent in affected cows in another report (14), although trauma was the expected cause. This may have been related to the chronicity of the condition. Previous studies have identified lameness as a key risk factor for rib and sternal swellings, likely due to impaired lying behavior on hard surfaces, which leads to rib injuries (1, 12, 23). Rib injuries have also been documented in slaughtered cattle that had collided with gates and other objects (62, 63), as well as in humans, where seatbelt-related injuries increase the risk of rib and sternal fractures (64–66). An analogous scenario is possible in dairy cattle during routine restraint in a hoof-trimming chute that uses only a chest belt or metal brackets to secure the thorax. Although trauma caused by inadequate fixation in a hoof-trimming chute has not been reported (PubMed search on 12 April 2025, keywords: claw trimming, rib fracture, cattle), it remains a potential risk for injury. Visible changes to the skin and hair coat after blunt trauma caused by objects with a smooth surface can be mild. Furthermore, the causative injury may have occurred long before the animal was examined. Thus, the risk posed by restraint in a hoof-trimming chute, as well as by gates and other objects in the housing environment, milking parlor, hoof trimming area, and slaughter facilities, should be examined in future studies.

### 3.5 Relevance of osteomyelitis of the ribs and sternum

All cows in this case series had severe diseases with a poor prognosis. They were lame and exhibited compromised health status, and three of them had a pain response when pressure was applied to the rib swellings. In all cases, discussions were held with the responsible farm personnel prior to euthanasia regarding the clinical findings, possible etiologies, and farm-specific risk factors. On-farm investigations were conducted, including systems-based analyses of potential environmental and management-related risk factors, as well as locomotion scoring and assessment of rib and sternal swellings. The diagnostic implications, potential causes, and possible consequences for animal health, welfare, and farm economics were discussed in detail with the responsible farm personnel. Previous studies reported rib swellings in 2.3 to 9.6% of the cows examined (1–3, 23), but EVT and OM of the ribs were not diagnosed, likely due to a lack of thorough examination of the cardiovascular system and the ribs and sternum. Reliable prevalence data on OM of the ribs or sternum in cattle are scarce. Endocarditis valvularis thromboticans occurred in 0.15% (67) of slaughtered cows and 9% of autopsied cattle (68). The difficulty in identifying cows with OM of the ribs and EVT raises animal welfare concerns, (13, 17–19), particularly as both rib and sternal lesions (10, 11, 14, 15) and EVT (69) are painful, chronic conditions with a poor prognosis. The clinically deteriorated, polymorbid condition of the cows in this report, along with the presence of decompensated EVT, warranted immediate euthanasia of all five cows (cow #4 at the follow-up examination). However, timely surgical intervention for the OM in cow #4 at the initial presentation might have potentially prevented the development of EVT. Successful therapy is contingent upon early detection and adequate intervention (31). To date, there are no treatment recommendations available for cows with OM of the ribs and sternum (PubMed search on 12 April 2025, keywords: cattle, osteomyelitis, rib, sternum, therapy). In human medicine, such cases have been successfully treated with extended antibiotic therapy, radical surgical intervention, including wound debridement and drainage, often combined with vacuum-assisted wound therapy, and intensive care support (30, 32, 33, 70). However, even in patients with well-healed, uncomplicated rib and sternum fractures, chronic pain has often been reported (71). In our case, such an intensive treatment approach, as described for human patients, would not have been feasible on a commercial dairy farm. However, in the future, early identification of such cases may allow for the development of practical, farm-adapted therapeutic options for cattle.

This case series highlights the importance of rib and sternal lesions and their secondary complications in cattle. The painful lesions and diagnostic challenges emphasize their impact on animal welfare. In addition to inspection and palpation of the ribs and sternum, future studies should include the various diagnostic steps outlined here for both individual cases and herd-level assessments. Early detection of OM of the ribs and sternum could, ideally, facilitate timely intervention and the development of practical treatment strategies, potentially reducing the long-term animal welfare concerns associated with these conditions.

## 4 Conclusion

Osteomyelitis (OM) of the ribs and sternum is an important animal welfare concern. It leads to chronic disease, may cause pain and suffering, and can be fatal. Clinical diagnosis is difficult in live animals, and thus, diagnosis may be delayed, jeopardizing the well-being of cattle. A thorough clinical examination, along with additional diagnostic tools—particularly ultrasonography and laboratory analyses—is required for diagnosing OM of the ribs and sternum and endocarditis valvularis thromboticans (EVT) in live cows. In the authors’ opinion, these diagnostic tools should be integrated into a standard examination protocol for cows with swellings associated with the ribs or sternum to ensure that OM and EVT are not overlooked. *Trueperella pyogenes* was the main pathogen in all the lesions examined. This pathogen was cultured using conventional sampling and culture techniques under field conditions. In addition to the factors discussed in the literature, trauma caused by environmental elements such as gates and poor fixation methods in hoof-trimming chutes should be considered potential causes of OM of the ribs and sternum.

## Data Availability

The original contributions presented in the study are included in the article/Supplementary material, further inquiries can be directed to the corresponding author.
