# Correction: Case Report: Clinical, laboratory, and pathological findings in cows with osteomyelitis of the ribs and sternum and endocarditis valvularis thromboticans

**DOI:** 10.3389/fvets.2025.1681013

**Published:** 2025-08-29

**Authors:** Frontiers Production Office

**Affiliations:** Frontiers Media SA, Lausanne, Switzerland

**Keywords:** osteomyelitis, endocarditis, Trueperella pyogenes, bacteriology, blood culture, ultrasonography, postmortem examination, cattle

The incorrect version of Supplementary Table 1 was erroneously published with the original version of this paper. The file has now been replaced.

The original version of this article has been updated.

