# Supplementary material for: Case Report: Clinical, laboratory, and pathological findings in cows with osteomyelitis of the ribs and sternum and endocarditis valvularis thromboticans
Source: Front Vet Sci. 2025 Jul 11;12:1589472. doi: 10.3389/fvets.2025.1589472 (PMC12292018; doi:10.3389/fvets.2025.1589472)
Supplement: Supplementary file 1 [file Table_1.docx]

Supplementary Material

The cows underwent a thorough clinical examination (21). The location, shape, and texture of the lesions associated with the ribs and sternum were assessed, and a tape measure was used to determine their size dorsoventrally and craniocaudally and lateral extension from the thoracic wall (21).

# Diagnostic procedures

## Ultrasonographic examination

B-mode echocardiography was carried out using a 4‑MHz sector transducer (MyLabDelta, Esaote) as described by Hollenberg (25). The ribs and sternum were examined using B-mode ultrasonography (MyLabDelta, Esaote) and a 10‑MHz linear transducer. The examination procedure for the latter was based on that described by Bitschnau (22) but modified for use in cattle. Changes were described according to Kofler (23). Swellings and effusions were examined for flow phenomena using sonopalpation (24).

## Sampling

Blood samples were collected from a jugular vein into a tube containing 2 ml glutaraldehyde, a tube containing 4 ml with ethylenediaminetetraacetate, and a 10 ml serum tube in all cows. Aseptic procedure was used to collect two 20 ml blood samples from a jugular vein, which were immediately placed in a polycarbonate blood culture bottle (BACT/ALERT® FA/FN Plus, BioMerieux) for aerobic and anaerobic blood culture. Fluid-filled cavities in the sternum and ribs were aspirated under ultrasound guidance using aseptic procedures (14 G cannula; 24). The aspirated fluid was placed in liquid AMIES medium (eSwab®, Copan Diagnostics Inc.).

## Laboratory diagnostics

The glutaraldehyde test (33) was carried out immediately after blood collection, and the serum blood sample was centrifuged at 1,100 x g for ten min at room temperature. Hematologic (ADVIA 120, Siemens Healthcare Diagnostic Products GmbH, Marburg) and clinical chemistry (Cobas c 311, Roche Diagnostics GmbH, Mannheim) analyses were done within 12 hours (h) of sampling. The blood culture bottles were incubated for a minimum of 48 h and a maximum of seven days at 37 ℃ and subcultured aerobically on Columbia blood agar, microaerobically on chocolate blood agar and anaerobically on Schaedler blood agar for 24 to 48 h. The fluid and tissue samples obtained during postmortem examination were refrigerated (4℃) and transferred to the same specialized laboratory for microbiologic examination within 24 h. In the laboratory, the samples were plated on Columbia blood agar, Columbia nalidixic acid blood agar, Schaedler blood agar, and chocolate blood agar and incubated for 24 to 72 h at 37℃ . Positive culture plates were further analyzed using MALDI-TOF(45).

## Gross and microscopic analysis

Postmortem examinations were performed on all cows. Tissue samples were collected from brain, lung, kidney, spleen, heart, liver and all lesions and placed in 4% neutral buffered formaldehyde for at least 24 h and embedded in paraffin by automatic paraffin infiltration (Donatello, DiaPath, Italy). Tissues with lesions were also stored as native samples at -20° C. Two to 4 µm sections were cut from Formalin-fixed paraffin-embedded (FFPE) – blocks, mounted on glass slides and stained with hematoxylin and eosin (H&E) stain according to standard operating protocols. The H&E-stained slides were examined using a light microscope (Olympus BX 53; Olympus, Life Sciences, Japan).

# Supplementary Figures and Tables

## Supplementary Figures


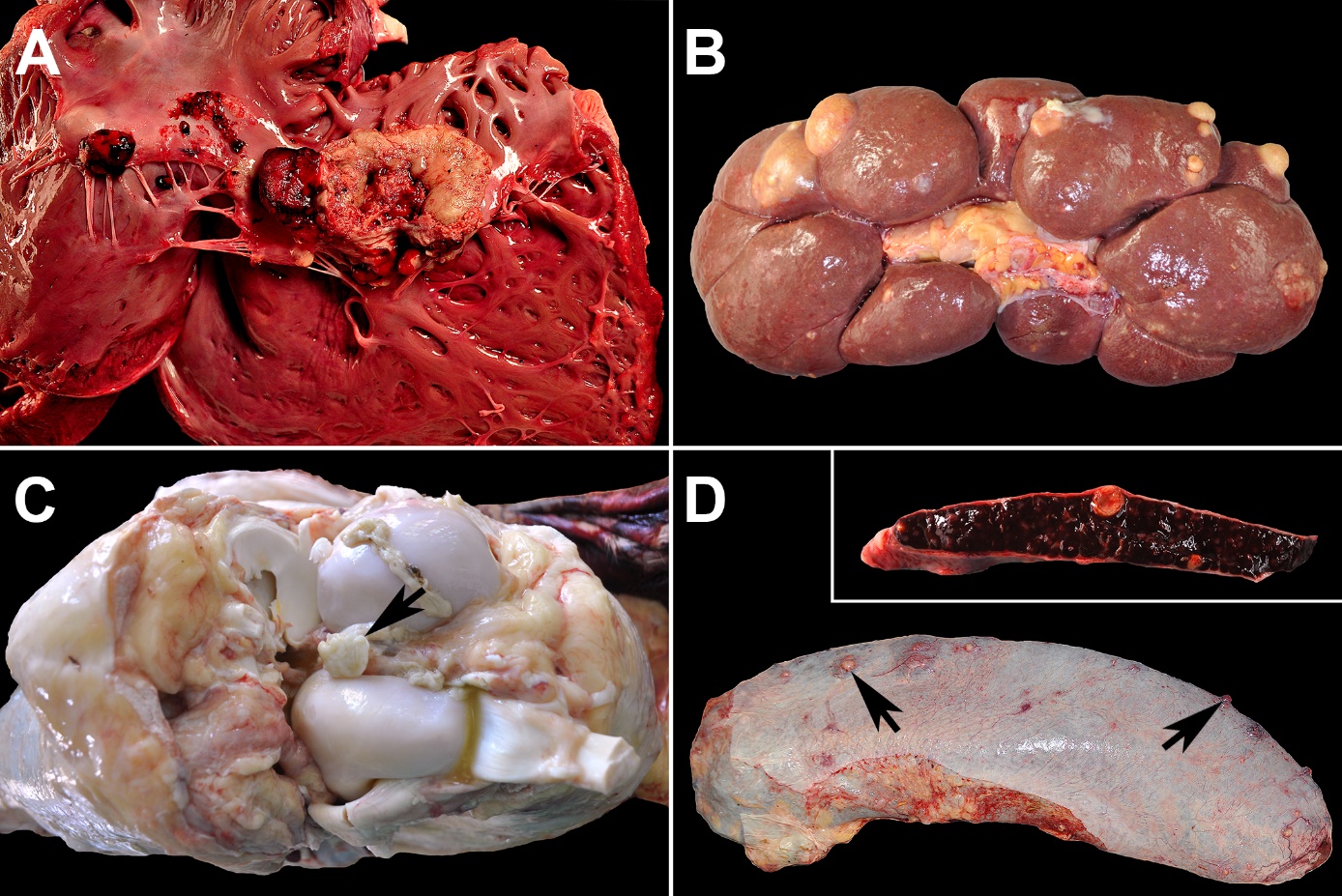


**Supplementary Figure 1:** **Embolic-metastatic lesions in different organs of cows: (A)** Severe endocarditis valvularis thromboticans of the tricuspid valve (cow #4). **(B)** Severe multifocal embolic suppurative nephritis (cow #4). **(C)** Moderate multifocal fibrino-suppurative gonitis (cow #3). **(D)** Mild multifocal embolic suppurative splenitis (arrows). Inset shows an encapsulated abscess in a cross‑section of this spleen (cow #5).

## Supplementary Tables

Supplementary Table 1: Number, herd average 305-day milk yield in kilograms (kg), herd average productive life span in lactations, environment, and lameness prevalence of dairy cows in the herds of origin

| **Farm** | **1** | **2** | **3** | **4** | **5** |
| --- | --- | --- | --- | --- | --- |
| **Number of dairy cows** | 401 | 475 | 787 | 830 | 2,866 |
| **305-day milk yield (kg)** | 9,418 | 9,501 | 11,301 | 9,916 | 8,381 |
| **Productive life span (no. of lactations)** | 2.7 | 2.9 | 3.3 | 3.2 | 2.9 |
| **Environment** | Free-stall barn | | | | |
|  | RM, SB | SB | SB | SB | RM |
| **Lameness prevalence (herd level)** | 42% | 25% | 30% | 24% | 60% |

RM = raised stall with rubber mattress; SB = deep straw bedding

Supplementary Table 2: Selected results of the clinical examination in five German Holstein cows with swellings associated with the ribs or sternum and endocarditis valvularis thromboticans

| **Cow** | **#1** | **#2** | **#3** | **#4** | **#5** |
| --- | --- | --- | --- | --- | --- |
| **Age in years** | 8.4 | 4.5 | 3.9 | 5.2 | 5.7 |
| **Days in milk** | 304 | 287 | 135 | 154 | 235 |
| **Body condition score** | 3 | 2.25 | 2.5 | IE: 2.25; FE: 1.75 | 1.75 |
| **Duration of illness in days** | 18 | 35 | 2 | IE: 1  FE: 52 | 25 |
| **Rectal temperature** | 38.9 °C | 37.8 °C | 38.6 °C | 38.3 °C | 39.4 °C |
| **Circulatory system** |  |  |  |  |  |
| Jugular veins | Distended | NAD | NAD | IE: NAD; FE: positive venous stasis test | Distended |
| Heart rate/min | 88 | 80 | 84 | IE: 88; FE: 120 | 108 |
| Heart auscultation | Pounding, 1^st^ HB indistinct, systolic murmur (MA: tricuspid valve) | Pounding | Pounding | FE: Pounding, 1^st^ HB indistinct, systolic murmur (MA: tricuspid valve) | Pounding, arrhythmia, 1^st^ HB indistinct, systolic murmur (MA: tricuspid valve) |
| **Ribs & sternum** | 4^th^ & 8^th^ ribs (L), elbow to sternum;  8^th^ rib (R), shoulder | 5^th^ - 7^th^ ribs (L), elbow to sternum | 9^th^ rib (L), sternum;  cranial third of sternum (R) | IE & FE: 8^th^ rib (L), elbow;  8^th^ rib (R), elbow | 8^th^ - 10^th^ ribs (L), elbow  8^th^ & 9^th^ ribs (R), elbow  13^th^ rib (R), shoulder |
| Localization  (body site, LM) |  |  |  |  |  |
| Dimension of lesion in cm (dv, cc, ml) | 8^th^ rib (L): 15, 12, 4;  8^th^ rib (R): 12, 10, 3 | 30, 20, 4 | n.a. | IE & FE: 15, 8, 3 (L)  10, 15, 5 (R) | 15, 15, 3 (L)  8^th^ & 9^th^ ribs (R): 15, 10, 2 |
| Consistency of swelling | 4^th^ & 8^th^ ribs (L): hard  8^th^ rib (R): tautly elastic | Hard | Hard | IE & FE: hard | Hard to tautly elastic |
| Sensitivity to pressure | NAD | Sensitive | NAD | IE: sensitive  FE: NAD | Sensitive |
| **Musculoskeletal system** |  |  |  |  |  |
| LD & leg | III of V HL | l of V HL & HR | ll of V HL | IE: ll of V HL & HR  FE: lll of V HL & HR | ll of V HL |
| Joints and tendon sheaths | Carpal joint FR & stifle joint HR & HL affected | Tendon sheaths of the deep flexor tendon HL & HR affected | Tendon sheaths of the deep flexor tendon HL & stifle joint HL affected | NAD | Stifle joint HL & HR affected |
| Further findings | Multiple abscesses |  |  | IE & FE: claw bandage HL & HR; abscesses in semitendinosus & semimembranosus muscles HL & HR | Claw bandage HL |

cc = craniocaudal; dv = dorsoventral; FE = follow-up examination; FR = right front limb; HL = left hind limb; HR = right hind limb; IE = initial examination; L = left; LD = lameness degree (26); LM = anatomical landmark; MA = maximum audibility; ml = mediolateral; n.a. = not available; NAD = nothing abnormal detected; R = right

Supplementary Table 3: Results and reference ranges (58) of the initial laboratory analyses (total leukocyte count, glutaraldehyde test, total protein, and albumin concentration) in five German Holstein cows with swellings associated with the ribs or sternum and endocarditis valvularis thromboticans

| **Cow** |  | **#1** | **#2** | **#3** | **#4** | | **#5** |
| --- | --- | --- | --- | --- | --- | --- | --- |
|  | **Reference range** |  |  |  | **IE** | **FE** |  |
| Total leukocyte count (×10^3^/µL) | 5 – 10 | 11.7 | 13.7 | 12.8 | 11.5 | 13.9 | 11.2 |
| Glutaraldehyde test (min) | >15 | 1 | 1 | 1 | 1 | 0.5 | 0.5 |
| Total protein concentration (g/l) | 60 – 80 | 108 | 98 | 104 | 107 | 97 | 103 |
| Albumin concentration (g/l) | 30 – 40 | 15 | 10 | 22 | 24 | 13 | 19 |

IE = initial examination; FE = follow-up examination

Supplementary Table 4: Results of bacteriologic testing of blood, endocardium, muscle, tendon sheath, joints, spleen, kidney, uterus, and the fluid-filled cavities associated with the ribs and sternum in five cows with swellings associated with the ribs or sternum and endocarditis valvularis thromboticans

| **Cow** | **#1** | **#2** | **#3** | **#4** | **#5** | **Positive cultures (%) per organ*** |
| --- | --- | --- | --- | --- | --- | --- |
| **Cavity rib / sternum** | *T. pyo* | *T.pyo* | *T.pyo* | IE & FE: *T.pyo* | *T.pyo* | 6 / 6 (100%) |
| **Blood** | No growth | | *T.pyo* | *T.pyo*  *Helcococcus ovis* | *T.pyo* | 3 / 5 (60%) |
| **Endocardium** | n.a. | *T.pyo* | No growth | *T.pyo* | *T.pyo*  *Strepto-coccus sp.* | 3 / 4 (75%) |
| **Other organs** | Stifle joint: *T.pyo* | n.a. | Tendon sheat: *T.pyo*  Stifle joint: *T.pyo* | Abscess: *T.pyo* | Uterus: *T.pyo*  Spleen: *T.pyo*  Kidney: *T.pyo* | 7 / 7 (100%) |
| **Positive cultures (%) per cow*** | 2 / 3 (67%) | 2 / 3 (67%) | 4 / 5 (80%) | 5 / 5 (100%) | 6 / 6 (100%) |  |

IE = initial examination; n.a. = not analysed; FE = follow-up examination, *T.pyo = Trueperella pyogenes,* * based on all examinations conducted

Supplementary Table 5: Gross lesions in the five cows

| **Cow** | **#1** | **#2** | **#3** | **#4** | **#5** |
| --- | --- | --- | --- | --- | --- |
| **Suppurative-pyogranulomatous costochondritis and osteomyelitis** | 13^th^ rib (R): ++, 4^th^ & 5^th^ sternebra: ++ | 5^th^ - 7^th^ rib (L), 4^th^ sternebra: +++ | 2^nd^ rib (R), 9^th^ rib (L): ++ | 8^th^ rib (R+L).: ++ | 8^th^ - 10^th^ rib (L) &  8^th^, 9^th^ & 13^th^ rib (R): +++ |
| **EVT: tricuspid valve** | ++ | +++ | +++ | +++ | ++ |
| **EVT: bicuspid valve** | + | + | ++ | + | + |
| **EVT, aortic valve** | - | - | - | - | +++ |
| **Embolic suppurative**  **myocarditis** | - | ++ | - | - | - |
| **Embolic suppurative nephritis** | Both kidneys: ++/+++ | Left kidney: ++ | Both kidneys: + | - | Both kidneys: +++ |
| **Embolic suppurative pneumonia** | ++ | ++ | ++ | - | ++ |
| **Pyogranulomatous arteriitis with thrombembolus** |  |  |  | Arteria pulmonalis dextra: +++ |  |
| **Embolic suppurative splenitis** | - | - | - | - | + |
| **Suppurative endometritis** | - | - | - | - | ++ |
| **Suppurative to fibrinosuppurative Arthritis** | Right carpal joint: +++  Both stifle joints: ++ | Both stifle joints: ++ | Left stifle joint: ++  Left tarsal joint +++ | - | Right stifle joint: ++ |
| **Clawsv** | - | lateral hind claw (L): + suppurative laminitis, pododermatitis, periostitis | - | hind claws (L+R): +++ suppurative ulcerative pododermatitis | hind claws (L): +++ suppurative ulcerative dermatitis digitalis |
| **Skin/subcutis** | Metacarpus (R): +++ suppurative Bursitis praecarpalis | Sternal region: + proliferative ulcerative dermatitis | - | - | Carpal region (L): +++ fibrinosuppurative, pyogranulomatous dermatitis |
| **Suppurative mastitis** | ++ | - | - | - |  |
| **Suppurative endometritis** | - | - | - | - | ++ |
| **Suppurative myositis** | - | - | - | M. semitendinosus, semimembranosus, bilat.: +++ | - |

bilat. = bilateral; EVT = endocarditis valvularis thromboticans; L = left; M. = musculus; R = right; + = mild; ++ = moderate; +++ = severe

# Supplementary Video

**Supplementary Video 1:** Transcutaneous B-mode ultrasound video (4‑MHz sector transducer, penetration depth 38 cm) from the left caudal long axis view of the heart of cow #4 at the follow-up examination: Tachycardia and hyperechoic circumferential, cauliflower-like mass with shadow artifacts attached to the tricuspid valve are shown. Aortic valve, peri-, epi- and myocardium are unremarkable.
